# The Role of Digital Health Literacy and Cultural Intelligence in Cultural Competence of Private Hospital Nurses: Cross-Sectional Study

**DOI:** 10.2196/97334

**Published:** 2026-09-22

**Authors:** Emad Shdaifat, Amira Alshowkan, Bilal Alsaaideh

**Affiliations:** 1 Community Nursing Department, College of Nursing, Imam Abdulrahman Bin Faisal University, King Faisal Road, Dammam, Eastern Province, 34212, Saudi Arabia, 966 544312234; 2 Dr. Sulaiman Al Habib Medical Group, Al Khober, Saudi Arabia

**Keywords:** cultural competency, cultural intelligence, digital health literacy, DHL, nurses, Saudi Arabia

## Abstract

**Background:**

As patient populations become increasingly diverse, nurses must develop cultural competence to provide effective and equitable care.

**Objective:**

This study aims to examine the predictors of cultural competence among nurses, specifically investigating the roles of digital health literacy (DHL) and cultural intelligence, while also assessing how demographic factors, competence levels, and DHL relate to culturally competent behaviors.

**Methods:**

A cross-sectional study was conducted with 296 nurses at a private hospital in Saudi Arabia. Data were collected using validated instruments to assess DHL, cultural intelligence, and cultural competence. Descriptive statistics, independent *t* tests, Pearson correlations, and hierarchical multiple linear regression analyses were performed using SPSS (IBM Corp) to evaluate the associations and predictors of cultural competence.

**Results:**

In a sample of 296 nurses, cultural intelligence was observed at a moderate level, present in 35.8% (n=106) of participants. Conversely, more than half of the participants (n=158, 53.4%) exhibited low DHL. The majority of the nurses (n=195, 65.9%) demonstrated moderate cultural competence. Education was the only demographic factor significantly associated with cultural competence, with nurses holding a Bachelor of Science degree scoring higher than those with a diploma (*P*=.003). Furthermore, cultural intelligence and DHL each showed a strong positive correlation with cultural competence (*P*<.001). Hierarchical regression analyses indicated that both cultural intelligence (β=.427; *P*<.001) and DHL (β=.315; *P*<.001) were significant predictors of cultural competence, jointly explaining 47.7% of the variance (adjusted *R*²=0.460; *P*<.001). Nationality also emerged as a significant predictor (β=.105; *P*=.03), whereas other demographic variables were not statistically significant. Cultural intelligence accounted for the largest proportion of explained variance (Δ*R*²=0.375), followed by DHL (Δ*R*²=0.051).

**Conclusions:**

Cultural competence among nurses is predominantly influenced by cultural intelligence and DHL, rather than by most demographic variables. Consequently, enhancing DHL and cultural intelligence may strengthen culturally competent practice. It is recommended that targeted educational and professional development interventions be implemented to elevate competence and support high-quality patient care across diverse health care settings.

## Introduction

In an increasingly globalized health care environment, cultural competence has become an essential component of high-quality nursing practice. It enables nurses to deliver care that respects patients’ cultural and religious values, beliefs, and practices, thereby improving patient outcomes, satisfaction, and health care equity [1]. In addition to ethnicity and language sensitivity, culturally competent nursing care requires sensitivity to patients’ religious beliefs and practices, particularly in multicultural health care settings. Religious values may influence health care decisions regarding informed consent, end-of-life care, organ donation, reproductive health, dietary restrictions, and acceptance of specific medical treatments [2-4]. Previous studies have emphasized that culturally responsive care requires nurses to understand and respect patients’ religious and spiritual beliefs, including those associated with Islam, Judaism, Christianity, and other faith traditions, while maintaining ethical and patient-centered practice [2-4]. These findings further highlight that cultural competence extends beyond communication skills to include religious and spiritual awareness in clinical decision-making [2].

As health care systems become more culturally diverse, nurses must possess the knowledge, skills, and attitudes necessary to communicate effectively and provide culturally responsive care. This includes recognizing how cultural and religious diversity influences patients’ preferences, expectations, and health care decisions. Recent international literature also highlights that workforce diversity, intercultural communication, and technology-enhanced health care are increasingly shaping culturally competent nursing practice [5].

Cultural competence is influenced by multiple individual and professional factors, including digital health literacy (DHL) and cultural intelligence [6]. DHL, defined as the ability to search for, evaluate, and apply health information using digital technologies, has become an essential competency for evidence-based nursing practice in technology-driven health care systems [7,8]. Emerging evidence further suggests that digital technologies, including AI-supported educational tools and culturally adapted digital resources, can strengthen nurses’ clinical decision-making and promote culturally competent care. Likewise, cultural intelligence, the capability to function effectively across different cultural contexts, enables nurses to adapt their communication and care to the needs of culturally diverse patients [9,10].

International studies have demonstrated that structured educational interventions incorporating experiential learning, intercultural communication, reflective practice, and technology-enhanced learning improve both cultural intelligence and cultural competence among nursing students and practicing nurses [9,11,12]. In addition, nurses with higher DHL are more likely to use evidence-based clinical guidelines, patient education resources, and culturally appropriate digital tools to deliver individualized care [13,14]. Park and Nahm [15] further reported that nurses’ use of point-of-care technologies is associated with greater confidence in clinical decision-making and participation in culturally sensitive evidence-based practice.

The importance of these competencies is particularly evident in Saudi Arabia, where a rapidly evolving health care system serves an increasingly multicultural patient population [5]. Previous studies conducted in Saudi Arabia have identified gaps in cultural competence among both nursing students and practicing nurses, largely attributed to limited exposure to transcultural education and culturally sensitive communication training [5,16]. Hamdan Alshammari and Alboliteeh [6] further emphasized that professional competence and cultural competence are closely interconnected, suggesting that both education and clinical experience contribute to culturally congruent nursing care.

Demographic characteristics such as age, educational level, and years of clinical experience may further influence cultural competence, DHL, and cultural intelligence [13,17]. Nurses with greater clinical experience often demonstrate stronger cultural competence due to prolonged exposure to diverse patient populations, whereas younger nurses may possess stronger DHL but require additional support in translating these digital skills into culturally responsive clinical practice [7,18-20].

Although previous studies have independently demonstrated that DHL and cultural intelligence contribute to cultural competence, most research has focused on educational interventions or specific dimensions of cultural competence, whereas limited empirical evidence has examined how DHL and cultural intelligence jointly predict cultural competence among practicing nurses, particularly within the Saudi health care context [1,8]. Furthermore, the extent to which demographic characteristics contribute to cultural competence alongside these factors remains insufficiently explored [6,14]. Addressing this gap may inform educational strategies and workforce development initiatives to strengthen culturally competent nursing care in increasingly diverse health care settings.

Therefore, the purpose of this study was to investigate the predictors of cultural competence among nurses by examining the roles of DHL and cultural intelligence while controlling for demographic variables and competence levels.

## Methods

### Design and Setting

A cross-sectional design was used for this study. The research was conducted in a private hospital situated in the Eastern region of Saudi Arabia. Registered nurses from various clinical units, including emergency, intensive care, medical-surgical, and outpatient departments, were invited to participate. These diverse clinical settings offered a suitable context for investigating DHL, cultural intelligence, and cultural competence within the framework of routine nursing practice. This study constitutes an in-depth analysis of an existing dataset previously used to test a structural model of culturally competent behavior. In the current analysis, a multiple regression approach was used to examine the individual predictive effects of DHL, cultural intelligence, and demographic variables on cultural competence behavior.

### Participants and Sampling

A convenience sampling method was used to recruit eligible registered nurses. The inclusion criteria specified that participants must be registered nurses engaged in direct patient care with a minimum of 6 months of clinical experience. Nurses occupying primarily administrative or managerial positions were excluded from the study due to their limited direct patient interaction. An a priori power analysis conducted using G*Power (Heinrich Heine University Düsseldorf) determined that a minimum sample size of 222 participants was necessary to identify a small-to-moderate effect size (*f*²=0.05) at a significance level of α=.05, with 80% statistical power in a multivariable regression model.

### Measures

DHL was evaluated using the eHealth Literacy Scale, an 8-item instrument designed to measure individuals’ perceived capacity to locate, assess, and apply online health information. Each item was rated on a 5-point Likert scale ranging from 1 (strongly disagree) to 5 (strongly agree) [21].

Cultural intelligence was measured using the 20-item Cultural Intelligence Scale, which encompasses metacognitive, cognitive, motivational, and behavioral domains. Responses were recorded on a 7-point Likert scale, with values ranging from 1 (strongly disagree) to 7 (strongly agree) [22].

Cultural competence was assessed through an 8-item behavioral scale derived from established instruments measuring cultural competence. The items targeted observable culturally responsive nursing behaviors and were rated on a 5-point scale, where 1 indicated “never” and 5 indicated “always” [23]. In the present study, all instruments exhibited excellent internal consistency reliability, with Cronbach α exceeding 0.95.

Demographic data included age, gender, marital status, education level (diploma vs Bachelor of Science [BSc] in Nursing), specialty, years of experience, and nationality. In Saudi Arabia, a nursing diploma is conferred upon completion of a 2‐ to 3-year program at a health institute, whereas a BSc in Nursing requires successful completion of a 4- to 5-year university degree.

### Data Collection Procedure

The survey was administered using QuestionPro (QuestionPro Inc) in February 2026. Access was restricted to invited participants via a unique link. Nurses were invited through hospital nursing departments via email and WhatsApp. Of the 411 nurses who viewed the survey, 296 completed it (participation rate of 72.0%). IP address checking and cookies were enabled to prevent duplicate submissions. Incomplete questionnaires were excluded from analysis. Responses completed in less than 1 minute were excluded to ensure data quality.

### Ethical Considerations

Ethical approval was secured from the Institutional Review Board of the participating hospital, Dr Sulaiman Al Habib Medical Group (RC26.02.08). Participation in the study was voluntary, and informed consent was obtained prior to the commencement of data collection. No identifiable information was gathered and all responses were treated with confidentiality. Data were stored securely and were accessible solely to the research team. The study complied with the ethical principles delineated in the Declaration of Helsinki.

### Data Analysis

Data were analyzed using SPSS (version 22; IBM Corp). Descriptive statistics were computed for demographic variables and study constructs. Categorical variables were summarized using frequencies and percentages, while continuous variables were reported as means and SDs.

Levels of cultural competence, cultural intelligence, and DHL were categorized using percentile-based cutoffs for descriptive purposes. Categories were established as low, moderate, and high through visual binning based on percentile distribution in SPSS. This methodology facilitated the empirical derivation of cutoffs, reflecting the natural distribution of scores within the sample and enabling meaningful interpretation and comparative analysis across constructs.

Independent samples *t* tests were used to explore differences in competence across various demographic groups. Pearson correlation analysis was performed to investigate relationships among the primary study variables. Hierarchical multiple linear regression was conducted to identify predictors of cultural competence. Demographic variables were entered in the first block, cultural intelligence in the second block, and DHL in the final block. Statistical significance was established at *P*<.05.

Prior to regression analysis, all assumptions were rigorously tested. Normality of residuals was assessed using the Shapiro-Wilk test and visual inspection of P-P plots. Linearity was confirmed through scatterplots of standardized residuals against predicted values. Homoscedasticity was assessed via visual inspection of residual plots, and independence of errors was confirmed using the Durbin-Watson statistic, which fell within an acceptable range. All assumptions were met. Multicollinearity was assessed using tolerance values. All tolerance values exceeded 0.10 (ranging from 0.511 to 0.944), indicating no serious multicollinearity among predictors.

## Results

Internal consistency was evaluated using Cronbach α for all study scales (N=296). The Cultural Intelligence Scale, consisting of 20 items, exhibited excellent reliability (α=0.969). The eHealth Literacy Scale, comprising 8 items, similarly demonstrated high internal consistency (α=0.959). Likewise, the Cultural Competence Scale, which also included 8 items, reflected strong reliability (α=0.957). The corrected item-total correlations for all scales exceeded 0.65, and the Cronbach α values did not significantly increase upon the deletion of any item, indicating robust homogeneity and stability of the items within each construct.

The study’s sample comprised 296 participants, with a predominance of females (n=285, 96.3%), while males constituted only 3.7% (n=11) of the cohort. More than half of the participants were single (n=171, 57.8%), whereas 42.2% (n=125) were married. The majority of respondents held a BSc degree (n=221, 74.7%), while 25.3% (n=75) possessed a diploma qualification. In terms of clinical specialty, the majority were employed in non–critical care unit settings (n=234, 79.1%), with 20.9% (n=62) working in critical care environments. Furthermore, most participants were non-Saudi (n=225, 76.0%), while 24.0% (n=71) identified as Saudi nationals.

The mean age of the participants was 30.36 (SD 5.8) years, with a median age of 29 (IQR 25-34) years and a range spanning 23 to 51 years, indicating a relatively young sample. The average years of professional experience was 4.97 (SD 4.8) years, with a median of 3 (IQR 1-7) years and a range of 1 to 24 years, suggesting moderate variability in clinical experience among participants (Table 1).

**Table 1.** Demographic characteristics of participants (N=296).

Variable and category	Participants
Gender	
Male, n (%)	11 (3.7)
Female, n (%)	285 (96.3)
Marital status	
Single, n (%)	171 (57.8)
Married, n (%)	125 (42.2)
Education	
Diploma, n (%)	75 (25.3)
BSc^a^, n (%)	221 (74.7)
Specialty	
Critical care unit, n (%)	62 (20.9)
Non–critical care unit, n (%)	234 (79.1)
Nationality	
Saudi, n (%)	71 (24.0)
Non-Saudi, n (%)	225 (76.0)
Age (years)	
Mean (SD)	30.36 (5.8)
Median (IQR; range)	29 (25-34; 23-51)
Experience (years)	
Mean (SD)	4.97 (4.8)
Median (IQR; range)	3 (1-7; 1-24)

a BSc: Bachelor of Science.

The mean cultural competence score among participants was 28.62 (SD 4.2), with the majority, 65.9% (n=195), classified as possessing moderate competence and 34.1% (n=101) exhibiting low competence. Regarding cultural intelligence, the mean score was 85.87 (SD 14.0), with participants relatively evenly distributed across the competency levels: 35.8% (n=106) categorized as moderate, 34.1% (n=101) as low, and 30.1% (n=89) as high. The average DHL score was 33.16 (SD 5.9), indicating that over half of the participants (n=158, 53.4%) were classified as having low DHL, while 18.6% (n=55) fell into the moderate category and 28.0% (n=83) were classified as high. Overall, while cultural competence and cultural intelligence scores were predominantly moderate, a significant proportion of nurses demonstrated low DHL, underscoring a critical area that requires targeted professional development (Table 2).

**Table 2.** Levels of cultural competence, cultural intelligence, and digital health literacy of participants (N=296).

Variable and category	Score, mean (SD)	Participants, n (%)
Cultural competence	28.62 (4.2)	
Low (≤27)		101 (34.1)
Moderate (28–32)		195 (65.9)
Cultural intelligence	85.87 (14.0)	
Low (≤81)		101 (34.1)
Moderate (82-96)		106 (35.8)
High (>96)		89 (30.1)
Digital health literacy	33.16 (5.9)	
Low (≤32)		158 (53.4)
Moderate (33-37)		55 (18.6)
High (>37)		83 (28.0)

Independent samples *t* tests were conducted to assess differences in cultural competence across various demographic groups. A statistically significant difference was identified solely in relation to education level. Participants holding a BSc degree (mean 29.06, SD 4.14) exhibited significantly higher competence scores compared to those holding a diploma (mean 27.36, SD 4.40; *t*_294_=−3.022; *P*=.003). No statistically significant differences in competence were detected based on gender, marital status, specialty, nationality, age category, or years of experience (all *P*>.05). These findings suggest that educational qualification is the only demographic factor associated with elevated cultural competence scores within this sample (Table 3).

**Table 3.** Differences in cultural competence across demographic groups.

Variable and group	Participants, n (%)	Cultural competence score, mean (SD)	*t* test (*df*)	*P* value
Gender			−0.425 (294)	.67
Male	11 (3.7)	28.09 (4.95)		
Female	285 (96.3)	28.65 (4.24)		
Marital status			−1.339 (294)	.18
Single	171 (57.8)	28.35 (4.38)		
Married	125 (42.2)	29.02 (4.09)		
Education			−3.022 (294)	.003
Diploma	75 (25.3)	27.36 (4.40)		
BSc^a^	221 (74.7)	29.06 (4.14)		
Specialty			0.805 (294)	.42
Critical care unit	62 (20.9)	29.02 (3.83)		
Non–critical care unit	234 (79.1)	28.53 (4.37)		
Nationality			0.490 (294)	.62
Saudi	71 (24.0)	28.85 (4.15)		
Non-Saudi	225 (76.0)	28.56 (4.31)		
Age (years)			−0.236 (294)	.81
≤29	157 (53.0)	28.57 (4.42)		
>29	139 (47.0)	28.69 (4.09)		
Experience (years)			−0.437 (294)	.66
≤3	156 (52.7)	28.53 (3.93)		
>3	140 (47.3)	28.74 (4.62)		

a BSc: Bachelor of Science.

Pearson correlation analysis revealed the presence of strong and statistically significant positive relationships among all study variables. Cultural intelligence exhibited a robust correlation with DHL (*r*=0.694; *P*<.001) and a moderate to strong correlation with cultural competence (*r*=0.637; *P*<.001). Furthermore, DHL showed a strong positive association with cultural competence (*r*=0.610; *P*<.001). These findings suggest that higher levels of cultural intelligence and DHL are associated with higher levels of cultural competence. All correlations were significant at the .01 level (2-tailed), indicating substantial interrelationships among the constructs (Table 4).

**Table 4.** Pearson correlations among cultural intelligence, digital health literacy, and cultural competence (N=296).

Variable	Cultural intelligence	Digital health literacy	Cultural competence
Cultural intelligence			
*r*	1	0.694	0.637
*P* value	—^a^	<.001	<.001
Digital health literacy			
*r*	0.694	1	0.610
*P* value	<.001	—	<.001
Cultural competence			
*r*	0.637	0.610	1
*P* value	<.001	<.001	—

a Not applicable.

The final model accounted for 47.7% of the variance in cultural competence (adjusted *R*²=0.460; *P*<.001). After controlling for demographic variables, both cultural intelligence (β=.427; *P*<.001) and DHL (β=.315; *P*<.001) emerged as significant positive predictors of cultural competence. Nationality also reached significance (β=.105; *P*=.03), while age, gender, marital status, education, specialty, and experience did not reach significance in the final model. Cultural intelligence accounted for the largest proportion of explained variance (Δ*R*²=0.375), followed by DHL (Δ*R*²=0.051). See Table 5 for breakdown by predictor variable and Table 6 for models.

**Table 5.** Predictor variable results for hierarchical multiple linear regression predicting cultural competence (N=296).

Predictor		B	SE	β	*t* test (*df*)	*P* value
Constant		6.53	2.979	—^a^	2.192 (286)	.03
Age		−0.02	0.052	−.027	−0.376 (286)	.71
Gender		0.358	0.987	.016	0.363 (286)	.72
Marital status		0.259	0.441	.03	0.587 (286)	.56
Education		0.61	0.434	.062	1.408 (286)	.16
Specialty		−0.053	0.483	−.005	−0.109 (286)	.91
Experience		0.027	0.064	.03	0.413 (286)	.68
Nationality		1.051	0.48	.105	2.19 (286)	.03
Cultural intelligence		0.13	0.019	.427	6.978 (286)	<.001
Digital health literacy		0.226	0.043	.315	5.261 (286)	<.001

a Not applicable.

**Table 6.** Hierarchical multiple linear regression predicting cultural competence (N=296).

Model	*R*	*R*²	Adjusted *R*²	Δ*R*²	*F* (*df*)	*P* value
(1) Demographics	0.226	0.051	0.028	—^a^	2.208 (7, 288)	.03
(2) + Cultural intelligence	0.653	0.426	0.41	0.375	26.642 (8, 287)	<.001
(3) + Digital health literacy	0.69	0.477	0.46	0.051	28.958 (9, 286)	<.001

a Not applicable.

## Discussion

### Principal Findings

This study examined predictors of cultural competence among nurses, focusing on DHL and cultural intelligence, and explored associations with demographic factors and competence levels. Cultural intelligence was identified at a moderate level. In contrast, over half of the participants demonstrated low DHL. Most nurses (n=195; 65.9%) displayed moderate cultural competence. Education was the only demographic factor significantly associated with cultural competence, with BSc holders scoring higher than diploma holders. Cultural intelligence and DHL were positively correlated with competence, and hierarchical regression revealed that both significantly predicted cultural competence, explaining a substantial portion of its variance.

The results showed that nurses’ cultural intelligence was moderate. This finding is consistent with previous studies reporting moderate levels of cultural intelligence among nursing students and health care professionals [9,10]. Higher levels have been observed among nurse educators, suggesting that professional experience and continuous professional development may enhance cultural intelligence [16]. The moderate levels observed in this study are likely due to a lack of formal education in cultural intelligence and limited opportunities for structured intercultural learning. In Saudi Arabia, nurses are often exposed to culturally diverse patients, which can lay the foundation for intercultural interactions; however, this exposure alone may not be sufficient to develop advanced cultural intelligence without targeted educational programs [1].

More than half of the participants had low DHL, suggesting difficulties accessing, appraising, and applying digital health information. This finding is consistent with previous studies reporting inadequate DHL among nursing students and nurses [7,8]. Other studies have also highlighted considerable variability in DHL and emphasized the need for systematic educational interventions [13]. Conversely, higher levels of DHL have been reported among nurses with higher educational qualifications and professional positions, indicating that educational and workplace factors can positively influence digital competency [14]. Low DHL levels in the current study may be explained by a lack of training in using digital platforms, limited institutional support, and factors such as low confidence, restricted access, and the rapid pace of technological change [15].

Most nurses had moderate levels of cultural competence, consistent with previous studies among practicing nurses and nursing students in Saudi Arabia [1,5]. Moderate levels of cultural competence have commonly been reported in settings with limited formal transcultural nursing education, whereas greater professional experience has been associated with higher levels of competence [6,16]. The findings of this study may be due to a combination of theoretical knowledge gained through education and practical experience with different patients. However, the absence of continuous, structured programs for cultural competence may limit further development toward higher levels of competence [11,12].

Among the demographic variables, education was the only factor that significantly predicted cultural competence, with BSc holders outperforming diploma holders. This finding supports previous evidence suggesting that higher educational attainment is associated with stronger professional and cultural competencies [6,14]. However, previous research has also indicated that clinical experience and continuing professional education may enhance cultural competence regardless of initial educational preparation [1]. The current study’s results are likely a reflection of the fact that BSc courses involve more transcultural topics, evidence-based practice, intercultural communication, and digital literacy, which, when combined, improve cultural competence [7,9].

Both cultural intelligence and DHL were positively correlated with cultural competence, suggesting that nurses with high adaptability to diverse cultural settings and strong digital literacy skills exhibit greater cultural competence. This finding is consistent with previous research demonstrating that cognitive-cultural capabilities and digital competencies jointly contribute to culturally responsive nursing practice [9,10,13]. Conceptually, cultural intelligence and DHL represent complementary competencies. Cultural intelligence enables nurses to understand, interpret, and adapt to patients’ cultural beliefs, values, and communication styles, whereas DHL equips nurses to locate, evaluate, and apply culturally appropriate health information and digital resources. Together, these competencies facilitate informed clinical decision-making, individualized patient care, and effective communication across diverse cultural contexts. Although technological competence is important, previous studies suggest that digital skills alone are insufficient to ensure culturally competent practice without the motivation and ability to adapt to patients’ cultural needs [15]. The results therefore indicate that nurses with high cultural intelligence can decode patients’ cultural messages and respond appropriately, while those with high DHL can effectively use digital resources to support culturally competent care [7,8].

Hierarchical regression analysis also showed that cultural intelligence and DHL were significant predictors of cultural competence, accounting for a large proportion of the variance in scores. This finding reinforces previous evidence that cognitive-cultural capabilities and digital competencies are both important determinants of culturally competent nursing practice [9,16]. More importantly, the results support the multidimensional nature of cultural competence, demonstrating that effective culturally competent care depends on both interpersonal adaptability and technology-related competencies. Cultural intelligence facilitates adaptive cross-cultural interactions, whereas DHL enables nurses to integrate evidence-based digital resources into culturally responsive care. Their combined contribution suggests that neither competency alone is sufficient; rather, culturally competent nursing practice emerges from integrating both human-centered and digital capabilities. Nurses with adaptive cultural intelligence and DHL are therefore more likely to implement evidence-based, culturally responsive interventions that improve the quality of patient care [12]. Although these individual competencies explained a substantial proportion of cultural competence, organizational factors such as leadership support, access to culturally appropriate digital resources, and continuing professional development may further strengthen culturally competent practice. Future research should examine these organizational influences using longitudinal and theory-driven study designs [1].

### Recommendations

Based on the results of this study, the following recommendations can be made. Nursing education programs should integrate cultural intelligence and DHL into undergraduate curricula and continuing professional development to strengthen nurses’ ability to deliver culturally competent care. Simulation-based intercultural communication training and workshops focusing on culturally sensitive digital health services should also be incorporated to enhance both cultural adaptability and effective use of digital health technologies. Additionally, health care organizations should establish institutional policies that promote equitable access to culturally appropriate digital resources, provide ongoing technical and educational support, and foster culturally inclusive work environments through leadership initiatives. Efforts should also be directed toward improving digital accessibility by addressing barriers such as limited technological confidence, inadequate access to digital resources, and unavailability of linguistically and culturally appropriate digital health information for diverse patient populations.

As AI and digital health technologies become increasingly integrated into health care, future educational and organizational initiatives should explore their role in supporting culturally competent nursing practice, including clinical decision support, culturally tailored patient education, and communication across multicultural health care settings. Strengthening these competencies has the potential to improve health care quality, patient safety, and patient satisfaction. For future studies, longitudinal and mixed-methods research is recommended to provide a more comprehensive understanding of the development of cultural competence over time. Future studies should also investigate additional individual and organizational factors, such as leadership support, organizational culture, digital infrastructure, and access to professional development, which may influence cultural intelligence, DHL, and cultural competence among nurses in Saudi Arabia.

### Limitations

Although this study identified significant predictors of cultural competence among nurses, it has some limitations. Data were collected from a single private setting, limiting generalizability, and the cross-sectional design prevents causal inferences. Additionally, reliance on self-reported questionnaires may introduce response bias. Future studies should use larger, more diverse samples, longitudinal designs, and objective measures to strengthen the findings.

### Conclusions

This study identified DHL and cultural intelligence as key predictors of nurses’ cultural competence. While cultural intelligence was moderate, over half of the participants had low DHL, and most showed moderate competence. Education was the only significant demographic factor, with BSc holders scoring higher. Both cultural intelligence and DHL significantly predicted competence, highlighting the need for health care institutions to provide supportive environments with culturally appropriate resources and technology to enhance culturally competent care.

## Abbreviations

BSc: Bachelor of Science
DHL: Digital Health Literacy
